# 2437 A prospective study of cancer clinical trial availability and enrollment among adolescents/young adults treated at a Children’s Hospital or Affiliated Adult Cancer Specialty Hospital

**DOI:** 10.1017/cts.2018.154

**Published:** 2018-11-21

**Authors:** Stefanie M. Thomas, Jemily Malvar, Henry Tran, Jared Shows, David R. Freyer

**Affiliations:** 1 Children’s Center for Cancer and Blood Diseases, Children’s Hospital Los Angeles, Los Angeles, CA, USA; 2 Department of Pediatrics, Keck School of Medicine, University of Southern California, Los Angeles, CA, USA; 3 Department of Pathology, University of Oklahoma Health Sciences Center, Oklahoma City, OK, USA; 4 Department of Pathology, Long Beach Memorial/Miller Children’s Hospital, Long Beach, CA, USA; 5 Department of Medicine, Keck School of Medicine, USC Norris Comprehensive Cancer Center, University of Southern California, Los Angeles, CA, USA

## Abstract

OBJECTIVES/SPECIFIC AIMS: Low cancer clinical trial (CCTs) enrollment may contribute to the poor survival improvement for adolescents and young adults (AYAs, aged 15–39 years) with cancer. Treatment site is thought to exacerbate this problem. This study evaluated whether differences in CCT availability explain lower CCT enrollment depending on treatment site for AYAs. METHODS/STUDY POPULATION: This prospective, observational cohort study was conducted at an academic children’s hospital and an adult cancer hospital, 2 affiliated sites within a NCI-designated Comprehensive Cancer Center over 13 months. In consecutive AYA patients newly diagnosed with cancer at both site, it was determined whether an appropriate CCT existed nationally, was available locally, and if enrollment occurred. The proportions of AYAs in these categories were compared by site using the χ^2^ test. RESULTS/ANTICIPATED RESULTS: Among 152 consecutive AYA patients, 68 and 84 were treated at the children’s hospital and adult cancer hospital, respectively. AYAs treated at the children’s hospital had similar CCT existence nationally compared with AYAs treated at the adult hospital [36/68 (52.9%) vs. 45/84 (53.6%), *p*=0.938]. However, a significantly higher percentage of children’s hospital treated AYAs than adult hospital treated AYAs had an available CCT [30/68 (44.1%) vs. 14/84 (16.7%), *p*<0.001]. Enrollment percentages were similarly low in both groups [8/68 (11.8%) vs. 6/84 (7.1%), *p*=0.327]. DISCUSSION/SIGNIFICANCE OF IMPACT: Significantly fewer AYAs treated at the adult hospital had a CCT available, but national existence was similar at both sites. This suggests that institutional barriers to opening CCT have more importance at adult centers.

